# The complex relation between obstructive sleep apnoea syndrome, hypogonadism and testosterone replacement therapy

**DOI:** 10.3389/frph.2023.1219239

**Published:** 2023-10-10

**Authors:** Andrea Graziani, Giuseppe Grande, Alberto Ferlin

**Affiliations:** Unit of Andrology and Reproductive Medicine, Department of Medicine, University of Padova, Padova, Italy

**Keywords:** OSAS, hypogonadism, testosterone, testosterone replacement therapy, obesity

## Abstract

Obstructive sleep apnoea syndrome (OSAS) is an under-recognized medical disease. The main risk factors for OSAS are male sex, older age, obesity, and metabolic syndrome, that are also associated with male hypogonadism (MH). Therefore, obesity has been classically identified as the most evident link between OSAS and MH. However, OSAS is *per se* linked to the development of MH by a combined effect of hypoxia, increased night-time awakenings, reduced sleep efficiency and fragmented sleep. Similarly, MH might represent a risk factor for OSAS, mainly related to sleep disturbances that are frequently associated with low testosterone. Data on testosterone replacement therapy (TRT) in patients with OSAS are limited. Nevertheless, TRT is generally contraindicated by guidelines in the presence of untreated or severe OSAS. TRT might in fact worse OSAS symptoms in different ways. Furthermore, OSAS has been proposed to be a risk factor for secondary polycythaemia and TRT might exacerbate polycythaemia. Therefore, TRT in hypogonadal men affected by untreated OSAS or severe OSAS should be considered with caution and in a personalised way. Nevertheless, the type and dosage of TRT should be considered, as short-term high-dose TRT might worsen OSAS, whereas long-term lower doses could eventually determine a clinical improvement of symptoms of OSAS. Here we reviewed the data on the association between OSAS, MH and TRT, including the opportunity of assessment of patients who develop signs and symptoms of OSAS during TRT by polysomnography.

## Introduction

### Obstructive sleep apnoea syndrome

Obstructive sleep apnoea syndrome (OSAS) represents a common and often under-recognized and under-diagnosed medical disease which is characterised by sleep-dependent pauses and reductions in airflow (1, 2). In particular, the sleep-dependent pauses may be complete (apnoeas) or partial (hypopnoeas), further resulting, among other consequences of OSAS, in hypoxemia and sleep fragmentation (3). OSAS has a prevalence of about 15% in men and 5% in women in the adult age (4). Other data show how about 34% and 17% of middle-aged men and women, respectively, are affected by OSAS (3), whose prevalence has been increasing during the past decades (5). The prevalence of OSAS is higher in patients with systemic diseases, such as hypertension, heart failure, coronary artery disease, metabolic alterations and stroke (2–4, 6). On the other hand, OSAS is associated with an increased risk of hypertension, atrial fibrillation, myocardial infarction, insulin resistance, and stroke (2).

The main risk factors for OSAS are male sex, older age and obesity (3). In particular, regarding the association between obesity and OSAS, the risk of OSAS correlates with the body mass index (BMI), and obesity is probably the most relevant risk factor for OSAS (3). Epidemiological data show that about 50% of obese patients are affected by OSAS (3). The clinical symptoms of OSAS include, among others, snoring, nocturnal polyuria, daytime sleepiness, morning headache, neurocognitive deficits, reduced libido, irritability, and depressive symptoms (2, 4–6). In addition, excessive daytime sleepiness may cause motor vehicle and work-related accidents (7). Clinical categorization of OSAS is based upon apnoea-hypopnoea index (AHI), obtained by the polysomnography, that represent the ratio between the number of apnoeas and hypopnoea per hour which identifies mild OSAS (5–15), moderate OSAS (15–30) and severe OSAS (>30). Moreover, also an AHI > 15 per hour in the absence of symptoms may be diagnostic of OSAS (3).

The treatment of choice for OSAS is the application of continuous positive air pressure (cPAP) (3, 8, 9). Furthermore, it is mandatory to treat the underlying pathophysiological factors, such as obesity, in order to improve the symptoms and the severity of OSAS (8). Other approaches include, among others, mandibular advancement devices, maxillofacial surgery, bariatric surgery in case of morbid obesity, hypoglossal nerve stimulation (3, 8). In addition, an interesting future pharmacological approach might be based on histamine 3-receptor antagonist/inverse agonists (10).

### OSAS and male hypogonadism

Male hypogonadism (MH) is defined as the failure of the testis to produce normal concentrations of testosterone and/or to produce a normal number of spermatozoa (11, 12). MH can be a primary disorder or a secondary one, resulting in testicular (or hypergonadotropic) or central (hypogonadotropic) hypogonadism, respectively, albeit combined forms may also occur (13). Moreover, another classification of MH distinguishes between organic—due to a permanent dysfunction—and functional hypogonadism—due to a reversible condition, and this is the typical form of the so-called late-onset hypogonadism (11). MH becomes increasingly prevalent in men over 40 or 50, but it might be underdiagnosed in clinical practice (14).

As seen above, the prevalence of OSAS is higher in patients with systemic diseases and even in patients affected by metabolic syndrome (MetS). MH is related to such diseases, in particular to diabetes, obesity and MetS (15, 16), creating an association between MH and obesity (17). Therefore, this association represents the first and major link between OSAS and MH, mediated by obesity itself. A recent meta-analysis, conducted to evaluate the association between OSAS and testosterone concentrations and considering 24 case-control studies with a total of 1,268 male patients and 745 male control individuals, found that serum testosterone concentrations in OSAS patients were significantly lower with respect to the control group, therefore suggesting a correlation between OSAS and serum testosterone concentrations (18). Another recent meta-analysis, conducted by Su et al. (19) considered 18 studies with 1,823 patients (1,119 with OSAS and 704 controls) and found an inverse correlation between OSAS and serum testosterone concentrations, independently from BMI and age, with the severity of OSAS also correlating with serum testosterone concentrations, which were notably reduced in patients with severe OSAS.

**Figure 1:**
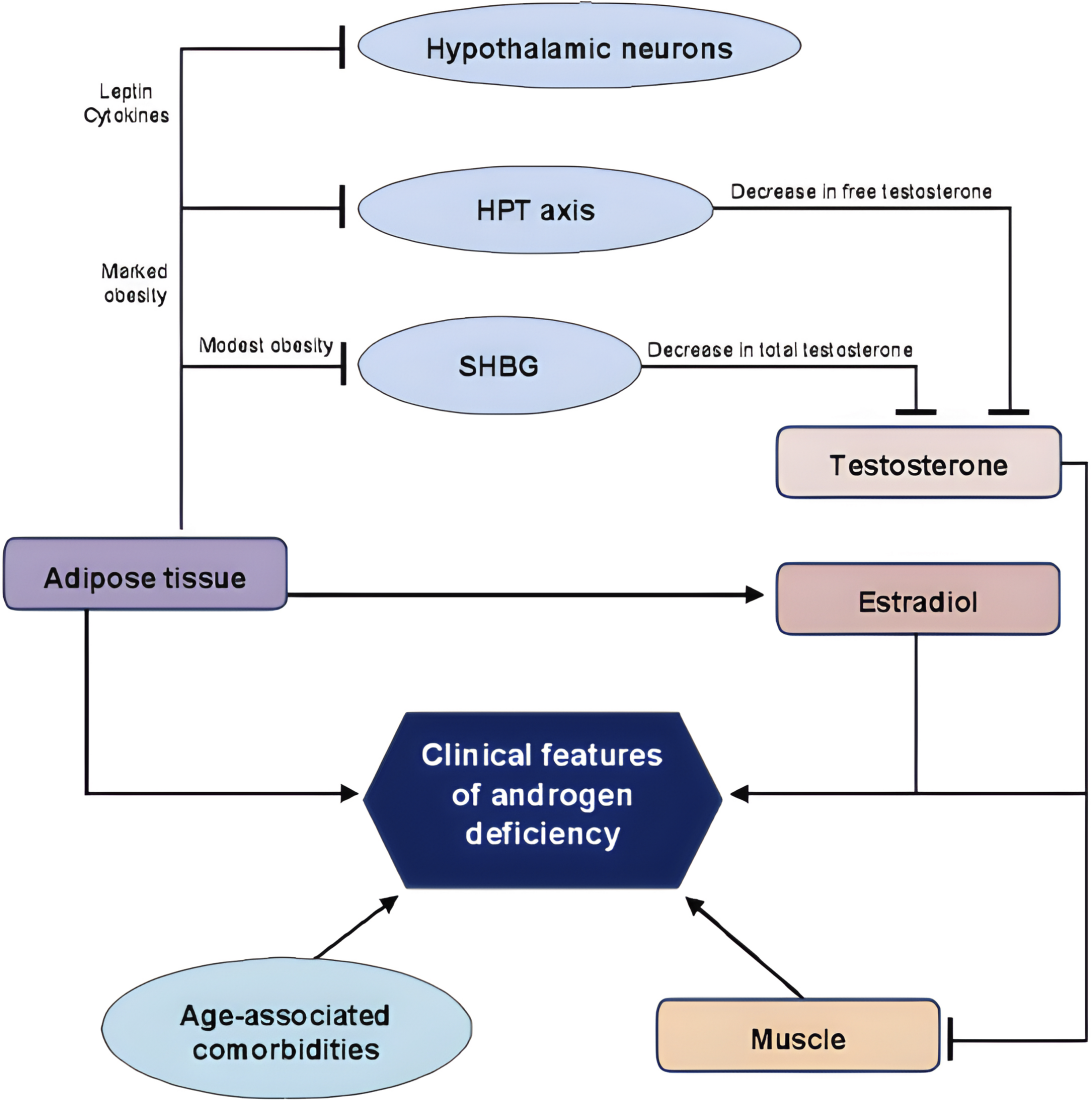
A schematic representation of the bidirectional association between adipose tissue and testosterone. HPT, hypothalamic pituitary axis; SHBG, sex hormone binding globulin. Lines: arrow line: stimulatory effect; non-arrow line: inhibitory effect. Adapted from Ref. (25).

The epidemiological connection between OSAS and obesity has already been discussed above. Obesity is a risk factor for OSAS because (i) it induces enlargement of structures surrounding the airway, contributing to upper airway narrowing; (ii) an excess of fat deposition is also observed under the mandible and in the tongue, soft palate and uvula; (iii) lung volumes are reduced in obese patients, further decreasing longitudinal tracheal traction forces and pharyngeal wall tension thus leading to the narrowing of the airway; (iv) obesity-related increase in leptin and leptin resistance might contribute itself to the genesis of OSAS (20, 21). The relation of obesity and MH is well known and bidirectional. Obese men have lower serum testosterone concentrations than non-obese men (22), due to modifications in sex hormone binding globulin (SHBG), increase in the aromatase enzyme activity of adipocytes (23, 24), low-grade systemic inflammation, increase in oestradiol concentrations, hyperinsulinemia/insulin resistance, and hyperleptinemia/lleptin resistance (22–24) (Figure 1).

**Figure 2:**
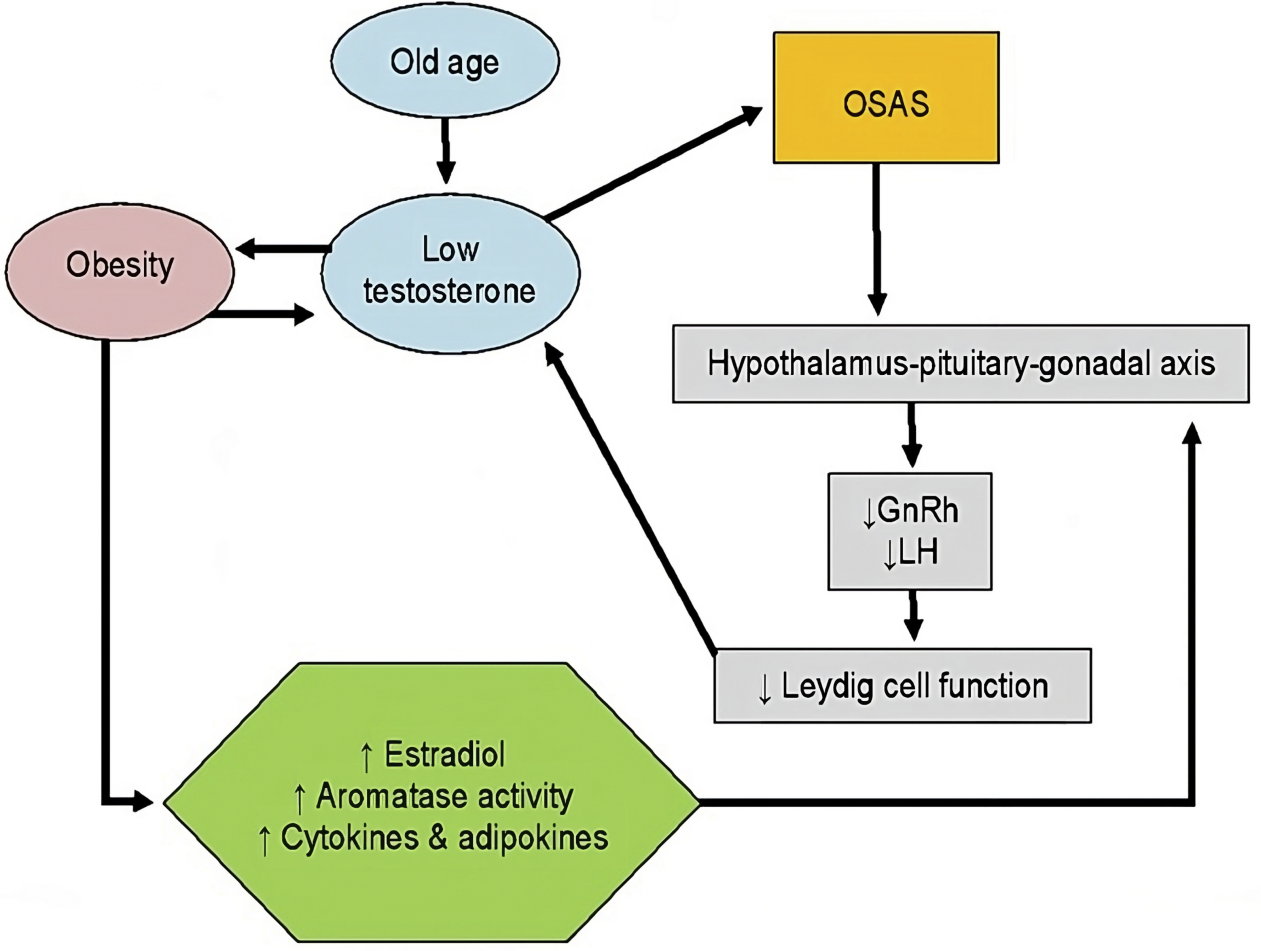
Association between testosterone, obesity and OSAS. GnRH, gonadotropin releasing hormone; LH, luteinizing hormone; OSAS, obstructive sleep apnoea syndrome. Lines: arrow line: stimulatory effect. Modified from Ref. (21).

Therefore, obesity induces a suppression of the hypothalamus–pituitary–gonadal axis (22, 24), representing one of the leading causes of secondary hypogonadism in men (23) with a biochemical picture characterised by normal or low concentrations of follicle-stimulating hormone (FSH) and luteinizing hormone (LH) and reduced serum testosterone concentrations (22–24). Given evidence of causal association between MH and obesity, MH may represent an additional risk factor for the development of OSAS. Furthermore, OSAS is *per se* linked to development of MH (26–28). In fact, OSAS has a direct inhibitory effect on pituitary function (26) lowering LH pulse amplitude and decreasing mean serum LH concentrations (24). In addition, OSAS’ sleep alterations lead to low total serum testosterone concentrations (24, 29) and to higher circulating leptin concentrations (24). Therefore, OSAS patients have reduced amounts of LH and testosterone, and therefore secondary hypogonadism, due to a pituitary-gonadal dysfunction induced by OSAS itself (27). This is caused by multiple and combined effects of hypoxia, increased night-time awakenings, reduced sleep efficiency and fragmented sleep (26, 28). In addition to this direct mechanism, OSAS reduces testosterone concentrations indirectly when associated with obesity, insulin-resistance or MetS (28) (Figure 2).

Therefore, as seen above, obesity is a risk factor for OSAS, and it is also related to hypogonadism in male patients (30). OSAS severity is related to total testosterone serum concentrations: in particular, a higher AHI score correlates with a lower serum testosterone concentration (26, 28, 31, 32). Furthermore, the severity of hypoxia during sleep is correlated with a reduction in testosterone concentrations (26, 32). While the quantity and quality of sleep have been linked to testosterone concentrations, other evidence may suggest the reversal association. For instance, it has been reported that patients with low serum testosterone concentrations have a decreased sleep efficiency and increased frequency of night-time awakenings (33). A future development might be the proposal of considering sleep disturbances as one of the symptoms for hypogonadism.

## Aim of the review

The relation between testosterone replacement therapy (TRT) and OSAS is more controversial. The aim of this review is to get more insight on this topic.

## Material and methods

We conducted a literature review on PubMed, up to December 2022, using the words “OSAS and hypogonadism”, “OSAS and testosterone”, “obstructive sleep apnea syndrome and testosterone”, “sleep apnea and testosterone”.

## Results

**Figure 3:**
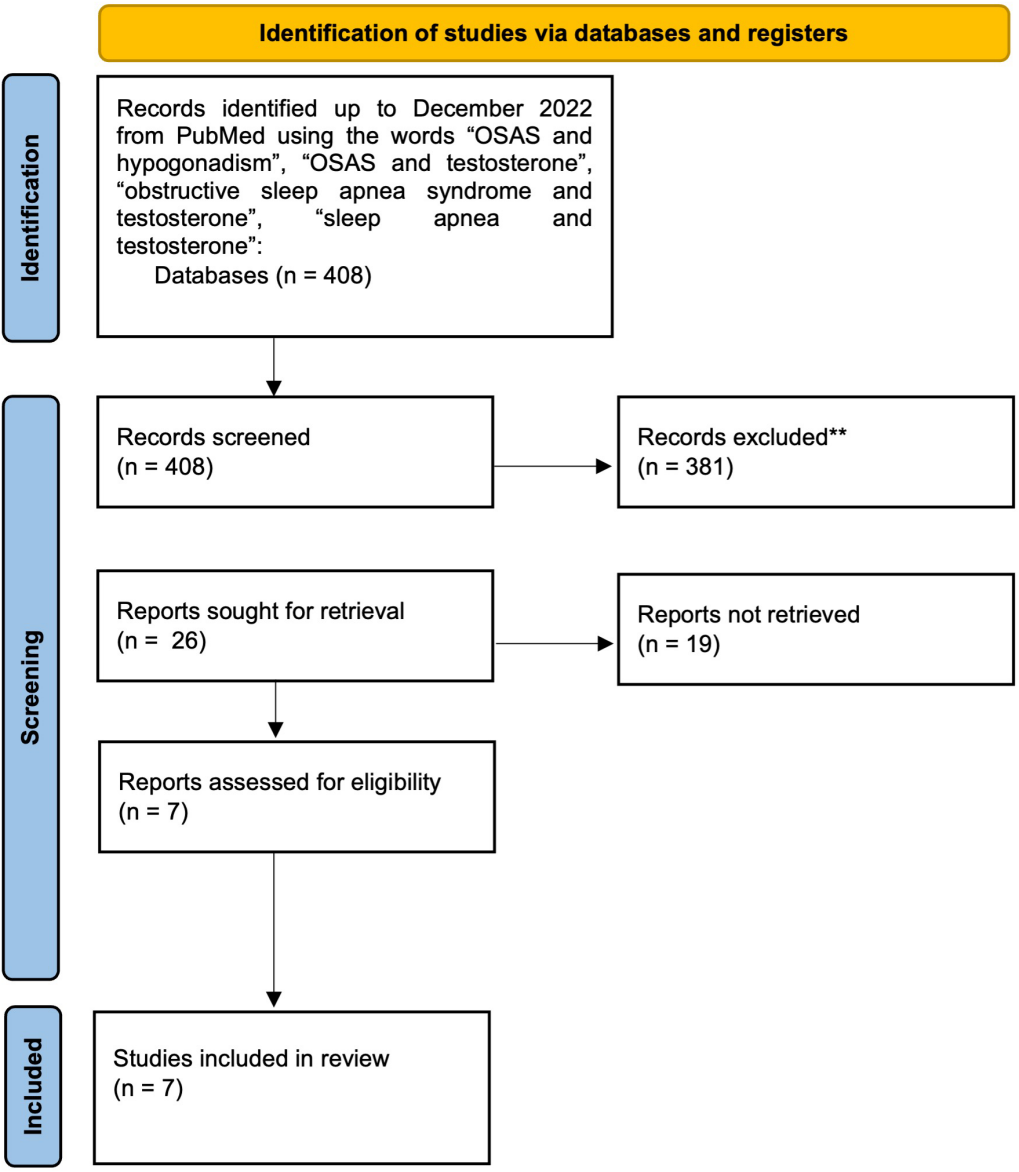
A schematic representation of the process of results’ collection regarding our literature search on PubMed. OSAS, obstructive sleep apnoea syndrome. *Reports excluded: non-English results, case reports, other studies/reviews, and studies regarding TRT and other conditions.

We collected the following results: “OSAS and hypogonadism” (6 results), “OSAS and testosterone” (14 results), “obstructive sleep apnea syndrome and testosterone” (151 results), “sleep apnea and testosterone” (235 results). After excluding non-English results, case reports, other studies/reviews, studies without available manuscripts and studies regarding TRT and other conditions, we included the clinical studies that reported the severity of OSAS pre/post TRT or changes in sleep functions or quality. The process of results’ collection is shown in Figure 3. We obtained 7 results, that are reported in Table 1.

**Table 1:** Summary of studies on OSAS and TRT.

Ref.	Type of study	Materials and methods	TT concentrations (mean)	Type of TRT	Length of TRT	Limitations proposed by authors	Results of authors	Conclusion of authors
Millman et al. (34)	Longitudinal	5 male patients in dialysis, evaluated by PSG performed both on and off weekly testosterone injections	///	Weekly im testosterone enanthate 250 mg	///	///	There was no change in sleep complaints or a decrease in the number of apnoeas and hypopneas off therapy	OSAS should be considered in symptomatic male dialysis patients, and it might not be solely related to testosterone administration
Liu et al. (35)	Randomized, double blind, placebo-controlled cross-over	17 patients (9 TRT, 8 placebo)	<15 nmol/L	Testosterone esters: 500 mg on week 0, 250 mg on week 1 and 2 (during phase 1); 500 mg on week 0, 250 mg on week 1 and 2 (during phase 2)	14 weeks	Treatment resulted in high serum testosterone concentrations and sleep and function were measured at the time of expected peak concentrations. Moreover, many of the subjects had a baseline testosterone within the normal range	Treatment reduced total time slept (about 1 h), increasing the duration of hypoxemia (about 5 min/night) and disrupted breathing during sleep	Short-term administration of high-dose testosterone shortens sleep and worsen OSAS
Hoyos et al. (36)	Randomized, double-blind, placebo-controlled, parallel group trial	67 obese men with severe OSAS (33 TRT, 34 placebo)	13.2 nmol/L for the testosterone group, 13.4 nmol/L for the placebo group	im testosterone undecanoate 1,000 mg or placebo at 0, 6 and 12 weeks.	18 weeks	///	Testosterone administration worsened the ODI by 10.3 events/h and nocturnal hypoxaemia at 7 weeks, whilst testosterone therapy did not alter ODI or O_2_ saturation at 18 weeks compared to placebo.	Testosterone therapy in obese men with severe OSAS mildly worsens sleep-disordered breathing in a time-limited manner, irrespective of initial testosterone concentrations
Killick et al. (25)	Randomised, double-blind, placebo-controlled, parallel group trial	10 TRT, 11 placebo Awake ventilatory chemoreflex testing was performed before, during and at the end of treatment Sleep and breathing was assessed by overnight PSG at 0, 7 and 18 weeks.	13.29 nmol/L for the testosterone group, 12.23 nmol/L for the placebo group	Testosterone undecanoate 1,000 mg or placebo at week 0, 6 and 12	18 weeks	The small number of participants. This study did not measure changes in upper airway anatomy nor the ventilatory chemoreflexes during sleep. Subjects were not specifically selected based on being hypogonadal	No significant differences were seen in ventilatory chemoreflexes between the two groups at 6 weeks or at 18 weeks. However, positive correlations were observed between changes in serum testosterone and hyperoxic ventilatory recruitment threshold and between changes in hyperoxic ventilatory recruitment threshold and time spent with oxygen saturations during sleep <90 at 6–7 weeks, but not at 18 weeks	Time-dependent alterations in ventilatory recruitment threshold may therefore mediate the time-dependent changes in sleep breathing observed with testosterone
Melehan et al. (37)	Randomized placebo-controlled	33 obese men with mild-severe OSAS treated with TRT, 34 controls	12.1 nmol/L for the testosterone group, 11.8 nmol/L for the placebo group	Im testosterone undecanoate 1,000 mg or placebo at baseline, week 6 and week 12.	18 weeks	///	In those with baseline testosterone concentrations <8 nmol/L, TRT increased vitality and reduced reports of feeling down and nervousness, whilst subjective sleepiness did not differ between the two groups	18 weeks of testosterone therapy increased sexual desire in obese men with OSAS independently of baseline testosterone concentrations whereas improvements in quality of life were evident only in those with testosterone concentrations <8 nmol/L
Cole et al. (38)	Retrospective matched cohort	3,422 male US military service members, during TRT 3,422 controls	///	80.4% of men used topical testosterone formulations, the remainder used a variety of injectable formulations, including a 7.13% of unclassified drug type	17 months (mean)	The possibility of unmeasured confounders, such as the specific aetiology of low testosterone concentrations, obesity, the lack of BMI. The lack of information on how OSAS was diagnosed The lack of information regarding the doses of TRT.	The risk of OSAS was higher in TRT users (2-year risk 16.5% in the TRT group vs. 12.7% in the control group.	The elevated risk of OSAS in men using TRT is noteworthy.
Lundy et al. (39)	Retrospective	474 men	<10.4 nmol/L	59% used Testopel, 22% intramuscular formulations and 19% topical formulations	About 48 months	Retrospective single-centre pilot study. All patients included in the study were treated by 1 single provider. It is difficult to ascertain from these data whether patients developed polycythaemia or OSAS first The small sample size limits the ability of data to ascertain whether specific TRT formulations are at higher risk for provoking polycythaemia and/or OSAS.	There was a positive association between polycythemic hypogonadal men and the concomitant diagnosis of OSAS. Moreover, at least 35% of this hypogonadal men was diagnosed with OSAS	The development of polycythaemia in hypogonadal men on TRT was associated with an increased prevalence of OSAS

im, intramuscular; ODI, oxygen desaturation index; OSAS, obstructive sleep apnoea syndrome; PSG, polysomnography; TRT, testosterone replacement therapy

The first study was published in 1985 and evaluated 5 male patients in dialysis using polysomnography and found no change in sleep complaints or a decrease in the number of apnoeas and hypopneas during and off therapy (34). The second study, conducted by Liu et al. (35), evaluating 17 patients, found that testosterone treatment reduced total time slept (about 1 h), increased the duration of hypoxemia (about 5 min/night) and disrupted breathing during sleep, thus leading to the conclusion that short-term administration of high-dose testosterone might shorten sleep and worsen OSAS. On the other hand, Hoyos Cm et al. reported that testosterone therapy in 67 obese men with severe OSAS might mildly worsen sleep-disordered breathing in a time-limited manner, irrespective of initial testosterone concentrations. In fact, testosterone administration worsened ODI by 10.3 events/h and nocturnal hypoxaemia at 7 weeks, whilst testosterone therapy did not alter ODI or O_2_ saturation at 18 weeks compared to placebo administration (36). The fourth study was conducted by Killick et al. (25) and reported no significant differences in ventilatory chemoreflexes between the testosterone and placebo group evaluated at 6 weeks and at 18 weeks. Melehan et al. (37) evaluated 33 obese men with mild-severe OSAS under TRT and 34 controls and found that in patients with baseline testosterone concentrations below 8 nmol/L TRT increased vitality and reduced reports of feeling down and nervousness, whilst subjective sleepiness did not differ between TRT and placebo group. In the sixth study (38) a higher risk of OSAS in TRT uses was noted (2-year risk 16.5% in the TRT group vs. 12.7% in the control group). Finally, Lundy SD et al. evaluated 474 hypogonadal men and reported that the development of polycythaemia in men on TRT was associated with an increased prevalence of OSAS (39).

Regarding the type of studies, just one is longitudinal (34), two are retrospective (38, 39), and few are randomised placebo-controlled clinical studies (25, 35–37). Mean testosterone concentrations at baseline are not always specified (34, 38) and, in some cases, it is even in the normal reference range (25, 36). Therefore, in some studies, testosterone therapy is not a real replacement therapy for hypogonadism, but rather a supplementation therapy. Type (38, 39) and length (34) of TRT are not uniform and not always clear. The number of patients is generally low, except for two studies (38, 39) that, on the other hand, have several limitations, such as the retrospective nature and the lack of information regarding the type of TRT. Furthermore, data on free testosterone are lacking. Finally, the primary aims of the studies are only sometimes directly related to the relation between OSAS and TRT, therefore creating further difficulties in the comprehension of this complex relation. In addition, there is a lack of long-term studies, which might prevent for example to possibly notice a positive effect of chronic low-dose TRT on OSAS symptoms, as suggested by some studies.

## Discussion

This review highlights that clinical data regarding TRT and OSAS are scarce and often lacking important clinical and/or biochemical data.

Although the data on TRT in patients with OSAS are limited and not uniform, as evidenced in this review, and there is a lack of convincing evidence that TRT causes and/or aggravates OSAS (25, 40–42), TRT is generally considered contraindicated in the presence of untreated or severe OSAS. The recent clinical practice guideline of the Italian Society of Andrology and Sexual Medicine (SIAMS) and the Italian Society of Endocrinology (SIE), regarding adult and late-onset hypogonadism, suggests not considering a treated OSAS as an absolute contraindication for TRT, albeit considering the lack of data regarding the role of TRT in men with OSAS (13). Other guidelines, surprisingly, such as the Society for Endocrinology guidelines for TRT in male hypogonadism, do not even mention OSAS among contraindication of TRT (43) whilst the Endocrine Society Clinical Practice Guideline (40) recommends against starting TRT in patients with untreated severe OSAS and considers the presence of the induction or worsening of obstructive sleep apnoea as an uncommon adverse with a weak association with TRT. Furthermore, a recent systematic review, conducted by Twitchell et al. (44) and evaluating the controversies about TRT, reported a positive association between TRT and OSAS. In addition, the European Academy of Andrology (EAA) guidelines on investigation, treatment and monitoring of functional hypogonadism in males does not consider the presence of OSAS as a contraindication for TRT but suggests evaluating the patient for hypoxia and sleep apnoea development during TRT (45). There are a variety of possible pathophysiological mechanisms by which TRT might exacerbate OSAS. TRT has been proposed to alter the central chemoreceptors (42, 46), therefore contributing to the development or worsening of OSAS via central mechanisms (26). A promising theory focuses on the role of androgens in neural response pathways to hypoxemia, but, according to the most recent data, the administration of testosterone has variable effects on ventilatory chemo-responsiveness and single unifying conclusion has not been reached yet. Notwithstanding, the evidence suggests that TRT might alter chemoreceptor stimulation thresholds or the ventilatory response to chemoreceptor stimulation by decreasing breathing stability (40).

One of the most debated parameters that seems to worsen after TRT is the morphological change in the upper airway. In particular, testosterone seems to impact the contraction of the airway dilator muscles and the following airway collapsibility. However, a consensus has not been found yet (26). A further connection between TRT and OSAS resides in the risk of polycythaemia. In fact, OSAS is a risk factor for secondary polycythaemia and TRT exacerbates polycythaemia in some patients (39, 40, 44). Furthermore, TRT may also induce higher metabolic rates, resulting in greater oxygen consumption leading to hypoxia (26). Finally, another possible explanation regarding the worsening of OSAS during TRT deals with a possible role of testosterone on the alteration of ventilatory response to hypoxia and hypercapnia, the alteration of serotonergic pathway and the reduction in time slept (26, 35).

Although the possible risks of TRT in subjects with OSAS are far to be clearly elucidated, it is important to note the benefit of restoring normal testosterone concentrations in men with hypogonadism. TRT is suggested in hypogonadal men for its clinical benefits regarding, among others, cardiometabolic risk, sexual function, bone metabolism and body composition (43, 47). Moreover, a recent clinical study (48) reported an improvement in sleep disturbance [defined as three or more points in question 4 of the ageing male symptoms (AMS) questionnaire] after 1 year of TRT in male affected by MH without OSAS. Therefore, while caution in TRT prescription should be maintained in patients with OSAS (38), a balanced and personalised counselling between risks and benefits should be discussed with the patients, taking also in consideration the possible co-morbidities and concomitant therapies. To this regard, it seems reasonable to delay TRT in hypogonadal men affected by untreated OSAS or severe OSAS. This review highlights also that modulation of type and dosage of TRT in the different patients is advisable. For example, it seems that short-term high-dose TRT could worsen OSAS, but these adverse effects could disappear with time (13, 42) and eventually determinate a clinical improvement of OSAS’ symptoms after 1 year of TRT (48). Therefore, a low dose TRT in the form of transdermal gel, which is more malleable if the clinical condition worsens, seems preferable. Finally, we suggest considering the assessment by polysomnography in patients who develop signs and symptoms of OSAS during TRT, with possible reduction or discontinuation of TRT if necessary.

Further considerations are necessary in this context. The severity of OSAS seems related to bone mineral density (BMD), as a recent prospective case–control study, enrolling 93 individuals (59 with OSAS and 34 as controls), reported lower BMD and vitamin D in patients with OSAS than controls, with a negative correlation between AHI and BMD (49). The risk of fracture and osteoporosis appears to be increased in patients with OSAS, probably due to different mechanisms, such as hypoxia, OSAS-related respiratory acidosis, leptin, OSAS-related comorbidities and, of course, hypogonadism (49). The assessment of bone status may become, in future, a parameter to evaluate when considering TRT in patients affected by OSAS, since the well-known positive effects of TRT in improving the bone status in hypogonadal patients (13, 50). On the other hand, the therapy of OSAS may improve the gonadal status and sexual functions. In fact, OSAS-related sleep fragmentation might disrupt the testosterone rhythm, with noteworthy attenuation of the nocturnal increase in testosterone (26). Studies showed a linear association between weight loss and increased serum testosterone concentrations in obese men (26). Few studies showed that the treatment of OSAS—both by surgery (51) and cPAP (52)—improve the gonadal function and testosterone concentrations. Nevertheless, other studies reported opposite results (26, 32, 46). In particular, a recent systematic review and meta-analysis (53) found that the cPAP use was not associated with a significant change in total testosterone concentrations, suggesting against the hypothesis of a direct interaction between OSAS and testosterone, albeit the conclusion that cPAP has no effect on testosterone concentrations is highly premature due to the low-quality available studies (54).

In addition, OSAS has been associated with altered HPG function and sexual dysfunction, manifested primarily as erectile dysfunction and decreased libido (22). Sexual functions of patients with OSAS and MH are thought to improve upon TRT, albeit this result should be confirmed in larger studies (37, 55). On the other hand, the therapy of OSAS might improve the OSAS-related erectile and sexual dysfunctions (26, 46). Finally, there are new concerns regarding testosterone hormone therapy in transgender males (assigned females at birth). In particular, Robertson et al. (56) reported two cases of transgender males in whom OSAS developed following initiation of testosterone therapy, with documented absence of OSAS before the sex affirming therapy.

### Perspectives and clinical messages

This manuscript underlines the need of an active collaboration between different specialists, including endocrinologists, general physicians, obesity physicians, otolaryngologists, pneumologist and sleep specialists. Furthermore, in the light of the evidence that OSAS represents a complex clinical condition—and not just a sleep disorder—and that nowadays multidisciplinary groups are created in order to offer a better care for complex patients, a multidisciplinary evaluation for patients with OSAS might be evaluated and even become mandatory in patients with OSAS and other complex diseases. Regarding the gonadal evaluation in patients with OSAS, albeit sleep disorders are not considered to be main signs/symptoms of hypogonadism (13), we would like to share the interplay between signs/symptoms that might be associated with both OSAS and MH, such as sexual dysfunction, low BMD, low motivation and vitality, poor concentration and memory, and even fatigue. Therefore, albeit for further studies are needed, a complete endocrine and metabolic evaluation in patients with OSAS might be suggested, in order to (i) evaluated endocrine-metabolic alterations caused by OSAS itself and (ii) evaluated possible endocrine-metabolic underlying disorders that might be even the cause of OSAS.

## Conclusion

In this review, we described the relation between OSAS and MH, which are two under-recognized and inter-connected medical disorders, with particular attention to TRT and OSAS. The analysis of the literature highlights the general lack of well-done studies and therefore the evidence is of low quality. We cannot confirm or reject the guidelines that suggest avoiding TRT in the presence of untreated or severe OSAS, as this appears a reasonable clinical practice. Indeed, some evidence suggests that short-time high-dose TRT might indeed worsen OSAS, whereas chronic low-dose TRT might improve OSAS. Notwithstanding, the therapy of OSAS might benefit the gonadal status and sexual functions. In the light of the few and sometimes inconclusive studies in this field, and therefore of the limited evidence and large grey areas, we wish for further studies and more active collaboration between endocrinologists and otolaryngologists.

## References

[1] Lee JJ Sundar KM. Evaluation and management of adults with obstructive sleep apnea syndrome. Lung. (2021) 199:87–101. 10.1007/s00408-021-00426-w 33713177

[2] Singh P Bonitati A. Obstructive sleep apnea syndrome—a review for primary care physicians and pulmonologists. R I Med J. (2021) 104:10–3. PMID: . 34437659

[3] Yeghiazarians Y Jneid H Tietjens JR Redline S Brown DL El-Sherif N Obstructive sleep apnea and cardiovascular disease: a scientific statement from the American heart association. Circulation. (2021) 144. 10.1161/CIR.0000000000000988 34148375

[4] Koka V De Vito A Roisman G Petitjean M Filograna Pignatelli GR Padovani D Orofacial myofunctional therapy in obstructive sleep apnea syndrome: a pathophysiological perspective. Medicina. (2021) 57:323. 10.3390/medicina57040323 33915707 PMC8066493

[5] Peppard PE Young T Barnet JH Palta M Hagen EW Hla KM. Increased prevalence of sleep-disordered breathing in adults. Am J Epidemiol. (2013) 177:1006–14. 10.1093/aje/kws342 23589584 PMC3639722

[6] Liu X Ma Y Ouyang R Zeng Z Zhan Z Lu H The relationship between inflammation and neurocognitive dysfunction in obstructive sleep apnea syndrome. J Neuroinflammation. (2020) 17:229. 10.1186/s12974-020-01905-2 32738920 PMC7395983

[7] Pollis M Lobbezoo F Aarab G Ferrari M Marchese-Ragona R Manfredini D. Correlation between apnea severity and sagittal cephalometric features in a population of patients with polysomnographically diagnosed obstructive sleep apnea. J Clin Med. (2022) 11:4572. 10.3390/jcm11154572 35956187 PMC9369523

[8] Antonaglia C Passuti G. Obstructive sleep apnea syndrome in non-obese patients. Sleep Breath. (2022) 26:513–8. 10.1007/s11325-021-02412-1 34324126 PMC9130173

[9] Santilli M Manciocchi E D’Addazio G Di Maria E D’Attilio M Femminella B Prevalence of obstructive sleep apnea syndrome: a single-center retrospective study. Int J Environ Res Public Health. (2021) 18:10277. 10.3390/ijerph181910277 34639577 PMC8508429

[10] Dauvilliers Y Verbraecken J Partinen M Hedner J Saaresranta T Georgiev O Pitolisant for daytime sleepiness in patients with obstructive sleep apnea who refuse continuous positive airway pressure treatment. A randomized trial. Am J Respir Crit Care Med. (2020) 201:1135–45. 10.1164/rccm.201907-1284OC 31917607 PMC7193861

[11] Duca Y Aversa A Condorelli RA Calogero AE La Vignera S. Substance abuse and male hypogonadism. J Clin Med. (2019) 8:732. 10.3390/jcm8050732 31121993 PMC6571549

[12] Grinspon RP Bergadá I Rey RA. Male hypogonadism and disorders of sex development. Front Endocrinol. (2020) 11. 10.3389/fendo.2020.00211 PMC7174651 32351452

[13] Isidori AM Aversa A Calogero A Ferlin A Francavilla S Lanfranco F Adult- and late-onset male hypogonadism: the clinical practice guidelines of the Italian Society of Andrology and Sexual Medicine (SIAMS) and the Italian Society of Endocrinology (SIE). J Endocrinol Invest. (2022) 45:2385–403. 10.1007/s40618-022-01859-7 36018454 PMC9415259

[14] Sizar O Schwartz J. Hypogonadism. In: Dulebohn S, editor. StatPearls. Treasure Island (FL): StatPearls (2022).

[15] Lotti F Marchiani S Corona G Maggi M. Metabolic syndrome and reproduction. Int J Mol Sci. (2021) 22:1988. 10.3390/ijms22041988 33671459 PMC7922007

[16] Louters M Pearlman M Solsrud E Pearlman A. Functional hypogonadism among patients with obesity, diabetes, and metabolic syndrome. Int J Impot Res. (2022) 34(7):714–20. 10.1038/s41443-021-00496-7 34775481

[17] Lamm S Chidakel A Bansal R. Obesity and hypogonadism. Urol Clin N Am. (2016) 43:239–45. 10.1016/j.ucl.2016.01.005 27132582

[18] Wang H Lu J Xu L Yang Y Meng Y Li Y Obstructive sleep apnea and serum total testosterone: a system review and meta-analysis. Sleep Breath. (2023) 27(3):789–97. 10.1007/s11325-022-02655-6 35904664

[19] Su L Meng Y Zhang S Cao Y Zhu J Qu H Association between obstructive sleep apnea and male serum testosterone: a systematic review and meta-analysis. Andrology. (2022) 10:223–31. 10.1111/andr.13111 34536053

[20] Drager LF Togeiro SM Polotsky VY Lorenzi-Filho G. Obstructive sleep apnea. J Am Coll Cardiol. (2013) 62:569–76. 10.1016/j.jacc.2013.05.045 23770180 PMC4461232

[21] Kuvat N Tanriverdi H Armutcu F. The relationship between obstructive sleep apnea syndrome and obesity: a new perspective on the pathogenesis in terms of organ crosstalk. Clin Respir J. (2020) 14:595–604. 10.1111/crj.13175 32112481

[22] Grossmann M. Hypogonadism and male obesity: focus on unresolved questions. Clin Endocrinol. (2018) 89:11–21. 10.1111/cen.13723 29683196

[23] Genchi VA Rossi E Lauriola C D’Oria R Palma G Borrelli A Adipose tissue dysfunction and obesity-related male hypogonadism. Int J Mol Sci. (2022) 23:8194. 10.3390/ijms23158194 35897769 PMC9330735

[24] Molina-Vega M Muñoz-Garach A Damas-Fuentes M Fernández-García J Tinahones F. Secondary male hypogonadism: a prevalent but overlooked comorbidity of obesity. Asian J Androl. (2018) 20:531. 10.4103/aja.aja_44_18 29974886 PMC6219298

[25] Killick R Wang D Hoyos CM Yee BJ Grunstein RR Liu PY. The effects of testosterone on ventilatory responses in men with obstructive sleep apnea: a randomised, placebo-controlled trial. J Sleep Res. (2013) 22:331–6. 10.1111/jsr.12027 23331844

[26] Kim S-D Cho K-S. Obstructive sleep apnea and testosterone deficiency. World J Mens Health. (2019) 37:12. 10.5534/wjmh.180017 29774669 PMC6305865

[27] Luboshitzky R Aviv A Hefetz A Herer P Shen-Orr Z Lavie L Decreased pituitary-gonadal secretion in men with obstructive sleep apnea. J Clin Endocrinol Metab. (2002) 87:3394–8. 10.1210/jcem.87.7.8663 12107256

[28] Molina FD Suman M de Carvalho TBO Piatto VB Taboga SR Maniglia JV Avaliação dos níveis séricos de testosterona em pacientes com síndrome da apneia obstrutiva do sono. Braz J Otorhinolaryngol. (2011) 77:88–95. 10.1590/S1808-86942011000100015 21340195 PMC9442381

[29] Wittert G. The relationship between sleep disorders and testosterone. Curr Opin Endocrinol Diabetes Obes. (2014) 21:239–43. 10.1097/MED.0000000000000069 24739309

[30] Fernandez CJ Chacko EC Pappachan JM. Male obesity-related secondary hypogonadism—pathophysiology, clinical implications and management. Eur Endocrinol. (2019) 15:83. 10.17925/EE.2019.15.2.83 31616498 PMC6785957

[31] Gambineri A Pelusi C Pasquali R. Testosterone levels in obese male patients with obstructive sleep apnea syndrome: relation to oxygen desaturation, body weight, fat distribution and the metabolic parameters. J Endocrinol Invest. (2003) 26:493–8. 10.1007/BF03345209 12952360

[32] Tančić-Gajić M Vukčević M Ivović M Marina LV Arizanović Z Soldatović I Obstructive sleep apnea is associated with low testosterone levels in severely obese men. Front Endocrinol. (2021) 12. 10.3389/fendo.2021.622496 PMC8350060 34381420

[33] Barrett-Connor E Dam T-T Stone K Harrison SL Redline S Orwoll E. The association of testosterone levels with overall sleep quality, sleep architecture, and sleep-disordered breathing. J Clin Endocrinol Metab. (2008) 93:2602–9. 10.1210/jc.2007-2622 18413429 PMC2453053

[34] Millman RP Kimmel PL Shore ET Wasserstein AG. Sleep apnea in hemodialysis patients: the lack of testosterone effect on its pathogenesis. Nephron. (1985) 40:407–10. 10.1159/000183509 4022209

[35] Liu PY Yee B Wishart SM Jimenez M Jung DG Grunstein RR The short-term effects of high-dose testosterone on sleep, breathing, and function in older men. J Clin Endocrinol Metab. (2003) 88:3605–13. 10.1210/jc.2003-030236 12915643

[36] Hoyos CM Killick R Yee BJ Grunstein RR Liu PY. Effects of testosterone therapy on sleep and breathing in obese men with severe obstructive sleep apnoea: a randomized placebo-controlled trial. Clin Endocrinol. (2012) 77:599–607. 10.1111/j.1365-2265.2012.04413.x 22512435

[37] Melehan KL Hoyos CM Yee BJ Wong KK Buchanan PR Grunstein RR Increased sexual desire with exogenous testosterone administration in men with obstructive sleep apnea: a randomized placebo-controlled study. Andrology. (2016) 4:55–61. 10.1111/andr.12132 26610430 PMC5035106

[38] Cole AP Hanske J Jiang W Kwon NK Lipsitz SR Kathrins M Impact of testosterone replacement therapy on thromboembolism, heart disease and obstructive sleep apnoea in men. BJU Int. (2018) 121:811–8. 10.1111/bju.14149 29383868

[39] Lundy SD Parekh NV Shoskes DA. Obstructive sleep apnea is associated with polycythemia in hypogonadal men on testosterone replacement therapy. J Sex Med. (2020) 17:1297–303. 10.1016/j.jsxm.2020.03.006 32307242

[40] Bhasin S Brito JP Cunningham GR Hayes FJ Hodis HN Matsumoto AM Testosterone therapy in men with hypogonadism: an endocrine society clinical practice guideline. J Clin Endocrinol Metab. (2018) 103:1715–44. 10.1210/jc.2018-00229 29562364

[41] Hanafy HM. Testosterone therapy and obstructive sleep apnea: is there a real connection? J Sex Med. (2007) 4:1241–6. 10.1111/j.1743-6109.2007.00553.x 17645445

[42] Payne K Lipshultz LI Hotaling JM Pastuszak AW. Obstructive sleep apnea and testosterone therapy. Sex Med Rev. (2021) 9:296–303. 10.1016/j.sxmr.2020.04.004 32636155

[43] Jayasena CN Anderson RA Llahana S Barth JH MacKenzie F Wilkes S Society for endocrinology guidelines for testosterone replacement therapy in male hypogonadism. Clin Endocrinol. (2022) 96:200–19. 10.1111/cen.14633 34811785

[44] Twitchell DK Pastuszak AW Khera M. Controversies in testosterone therapy. Sex Med Rev. (2021) 9:149–59. 10.1016/j.sxmr.2020.09.004 33309270

[45] Corona G Goulis DG Huhtaniemi I Zitzmann M Toppari J Forti G European Academy of Andrology (EAA) guidelines on investigation, treatment and monitoring of functional hypogonadism in males. Andrology. (2020) 8:970–87. 10.1111/andr.12770 32026626

[46] Burschtin O Wang J. Testosterone deficiency and sleep apnea. Sleep Med Clin. (2016) 11:525–9. 10.1016/j.jsmc.2016.08.003 28118875

[47] Thirumalai A Berkseth KE Amory JK. Treatment of hypogonadism: current and future therapies. F1000Res. (2017) 6:68. 10.12688/f1000research.10102.1 28149506 PMC5265703

[48] Shigehara K Konaka H Sugimoto K Nohara T Izumi K Kadono Y Sleep disturbance as a clinical sign for severe hypogonadism: efficacy of testosterone replacement therapy on sleep disturbance among hypogonadal men without obstructive sleep apnea. Aging Male. (2018) 21:99–105. 10.1080/13685538.2017.1378320 28920756

[49] Sadaf S Shameem M Siddiqi SS Anwar S Mohd S. Effect of obstructive sleep apnea on bone mineral density. Turk Thorac J. (2021) 22:301–10. 10.5152/TurkThoracJ.2021.20051 35110247 PMC8975338

[50] Corona G Vena W Pizzocaro A Giagulli VA Francomano D Rastrelli G Testosterone supplementation and bone parameters: a systematic review and meta-analysis study. J Endocrinol Invest. (2022) 45:911–26. 10.1007/s40618-021-01702-5 35041193

[51] Santamaria JD Prior JC Fleetham JA. Reversible reproductive dysfunction in men with obstructive sleep apnoea. Clin Endocrinol. (1988) 28:461–70. 10.1111/j.1365-2265.1988.tb03680.x 3145819

[52] Grunstein RR Handelsman DJ Lawrence SJ Blackwell C Caterson ID Sullivan CE. Neuroendocrine dysfunction in sleep apnea: reversal by continuous positive airways pressure therapy. J Clin Endocrinol Metab. (1989) 68:352–8. 10.1210/jcem-68-2-352 2493027

[53] Cignarelli A Castellana M Castellana G Perrini S Brescia F Natalicchio A Effects of CPAP on testosterone levels in patients with obstructive sleep apnea: a meta-analysis study. Front Endocrinol. (2019) 10. 10.3389/fendo.2019.00551 PMC6712440 31496991

[54] Liu PY Reddy RT. Sleep, testosterone and cortisol balance, and ageing men. Rev Endocr Metab Disord. (2022) 23:1323–39. 10.1007/s11154-022-09755-4 36152143 PMC9510302

[55] Zhuravlev VN Frank MA Gomzhin AI. Sexual functions of men with obstructive sleep apnoea syndrome and hypogonadism may improve upon testosterone administration: a pilot study. Andrologia. (2009) 41:193–5. 10.1111/j.1439-0272.2008.00914.x 19400854

[56] Robertson BD Lerner BS Collen JF Smith PR. The effects of transgender hormone therapy on sleep and breathing: a case series. J Clin Sleep Med. (2019) 15:1529–33. 10.5664/jcsm.7992 31596219 PMC6778344

